# Visualization of the renal vein filled with contrast agent may indicate the renal vein injury during percutaneous nephrolithotomy: two case reports

**DOI:** 10.1186/s12894-021-00868-7

**Published:** 2021-08-06

**Authors:** Xiao-Feng Chen, Jian-Jun Zhou, Jian-Ming Sun, Guo-Can Cao, Yi-Hua Zou, Wang-Long Deng

**Affiliations:** 1 Department of Urology, The First People’s Hospital of Chenzhou, 102 Luojiajing, Chenzhou, 423000 Hunan People’s Republic of China; 2 Department of Urology, The First Clinical College of Xiangnan University, 102 Luojiajing, Chenzhou, 423000 Hunan People’s Republic of China; 3 Department of Urology, The Fourth People’s Hospital of Chenzhou, Chenzhou, Hunan People’s Republic of China

**Keywords:** Nephrostomy, Percutaneous, Tube, Complication, Intravenous

## Abstract

**Background:**

Intravenous misplacement of a nephrostomy tube is a rare complication of percutaneous nephrolithotomy (PCNL) or percutaneous nephrostomy. The mechanism of misplacement of a nephrostomy tube into the vascular system is seldom investigated. One type of the possible mechanism is that the puncture needle penetrates a major intrarenal tributary of the renal vein and enters the collecting system. However, the guidewire is located outside the collecting system near the large branches of renal vein or perforates into the renal vein. The dilation is performed and causes a large torn injury. Subsequently, the nephrostomy tube is placed inside the vessel when radiological monitoring is not used. However, there is no imaging evidence and the scene of procedure is not demonstrated. This paper reports two cases of visualization of the renal vein filled with contrast agent during PCNL. The findings may be good evidence to support the step of renal vein injury in patients with intravenous nephrostomy tube misplacement.

**Case presentation:**

We presented two cases with visualization of the renal vein filled with contrast agent during PCNL. In the process of injecting the contrast agent through the puncture needle, we could see the renal vein. Moreover, it was identified that the puncture needle tip was not on the optimal position. The position of puncture needle tip lay outside the collecting system, which was close to the calyceal infundibulum and branches of renal vein.

**Conclusions:**

Visualization of the renal vein filled with contrast agent may be good evidence to verify the renal vein injury in patients with intravenous nephrostomy tube misplacement during PCNL or percutaneous nephrostomy. The suboptimal location of the puncture needle tip and visualization of the renal vein filled with contrast agent indicate the renal vein injury. One type of mechanism of intravenous nephrostomy tube misplacement is as following. Firstly, the guidewire stays outside the collecting system. Subsequently, dilatation directed by the guidewire results in the injury of the vein. Then, the nephrostomy tube migrates into the venous system due to prompt tube inserting and the direction of the sheath and/or the guidewire to the injured vein.

## Background

Intravenous misplacement of a nephrostomy tube is a rare event of percutaneous nephrolithotomy (PCNL) or percutaneous nephrostomy (PCN) [1–6]. Limited literatures have reported the misplacement of a nephrostomy tube in the renal vein, inferior vena cava (IVC) or atrium. The mechanisms of misplacement of a nephrostomy tube into the vascular system are also seldom investigated. One type of the possible mechanism is that the puncture needle penetrates a major intrarenal tributary of the renal vein and enters the collecting system. However, the guidewire is located outside the collecting system near the large branches of renal vein or perforates into the renal vein. The dilation is performed and causes a large torn injury. Subsequently, the nephrostomy tube is placed inside the vessel when radiological monitoring is not used [6, 9]. However, there is no imaging evidence and the scene of procedure is not demonstrated.

In the present study, we report two cases of visualization of the renal vein filled with contrast agent during PCNL at our centers. The findings may be good evidence to support the step of renal vein injury in patients with intravenous nephrostomy tube misplacement.

## Case presentation

**Fig. 1:**
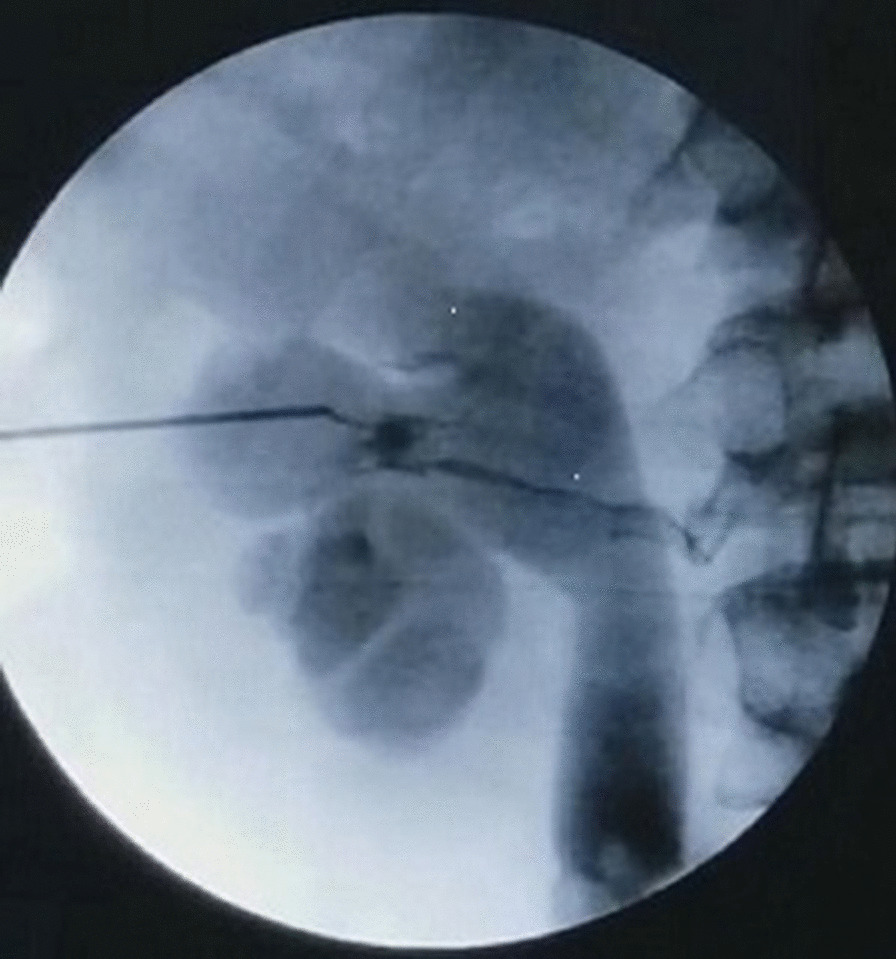
The radiograph of the kidneys, ureters and bladder revealing the left renal vein, the collecting system, and the suboptimal position of the puncture needle tip

**Fig. 2:**
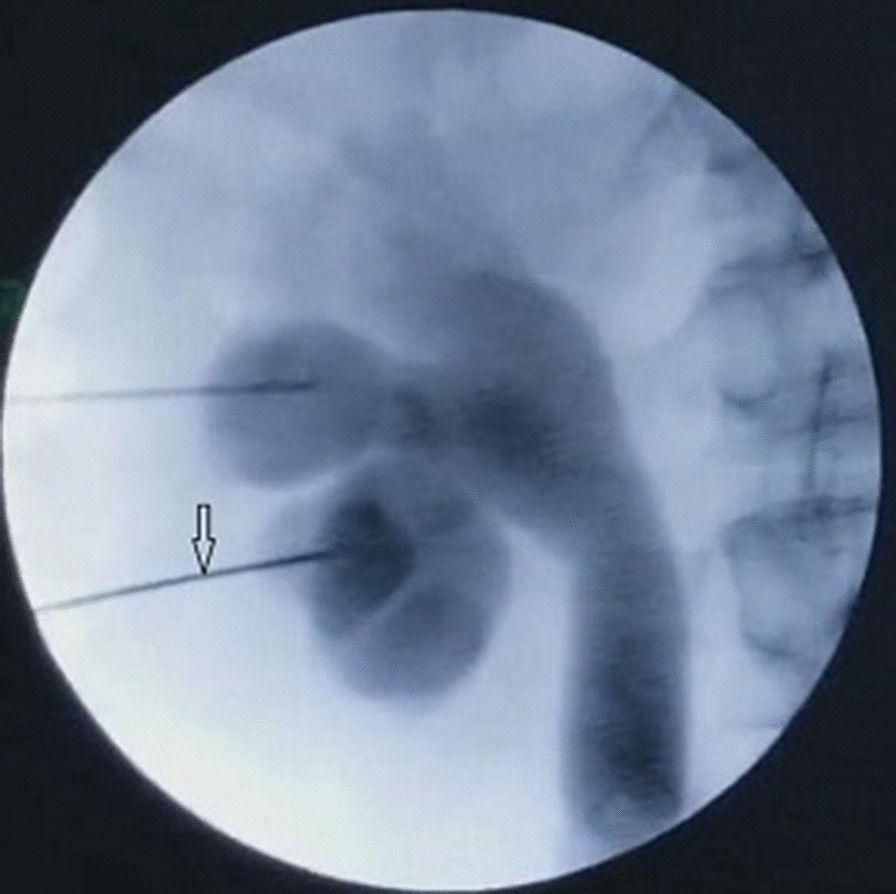
The radiograph of the kidneys, ureters and bladder revealing the collecting system, without visualization of the renal vein by injection of contrast agent through the latter puncture needle (arrow)

Patient 1 was a 45-year-old man who presented with left flank pain. His medical history indicated that he had renal stone. His physical examination was positive for tenderness upon palpation of his left flank. The initial laboratory evaluation showed normal. The computed tomography (CT) scan of abdomen and pelvis showed left moderate hydronephrosis and an upper ureteral stone was measured at 1.3 cm. Our patient was diagnosed with left upper ureteral stone with moderate hydronephrosis. The procedure of PCNL was carried out. A ureteral catheter was inserted retrogradely into the left pelvic under ureteroscopy. The puncture target calix was the middle posterior calix. The puncture site of the target calyceal fornix was localized under C-arm radiological monitoring using contrast agent injected through ureteral catheter, producing a retrograde pyelography (RP). An 18 gauge, 2 piece entry needle was used. Clear urine was seen when the needle stylet was removed. When we injected the contrast agent into his left collecting system through the puncture needle, we were able to see his left renal vein (Fig. 1). Moreover, we could identify that the location of the puncture needle tip was not optimal, the reason was that the puncture needle tip was located outside the collecting system near the calyceal infundibulum. At the moment, the patient had a hemodynamic stability and the puncture needle was maintained in its original place. Additional lower pole calix puncture was performed under radiological monitoring. Clear urine was also seen when the needle stylet was removed. Injection of contrast agent in his left collecting system through the later puncture needle showed the collecting system, without visualization of the renal vein (Fig. 2). In the process of injecting saline into his left collecting system by ureteral catheter, we were able to see the light red drips through the former puncture needle, and the clear drips through the latter puncture needle. The former puncture needle was pulled out and simultaneous PCNL was performed. An 18 gauge, 2 piece entry needle was advanced into the lower pole calix under fluoroscopy guidance. A zebra guidewire was inserted into the collecting system. The tract was dilated with fascial dilators to accommodate an 18 French sheath. The renal calculus was removed uneventfully.

**Fig. 3:**
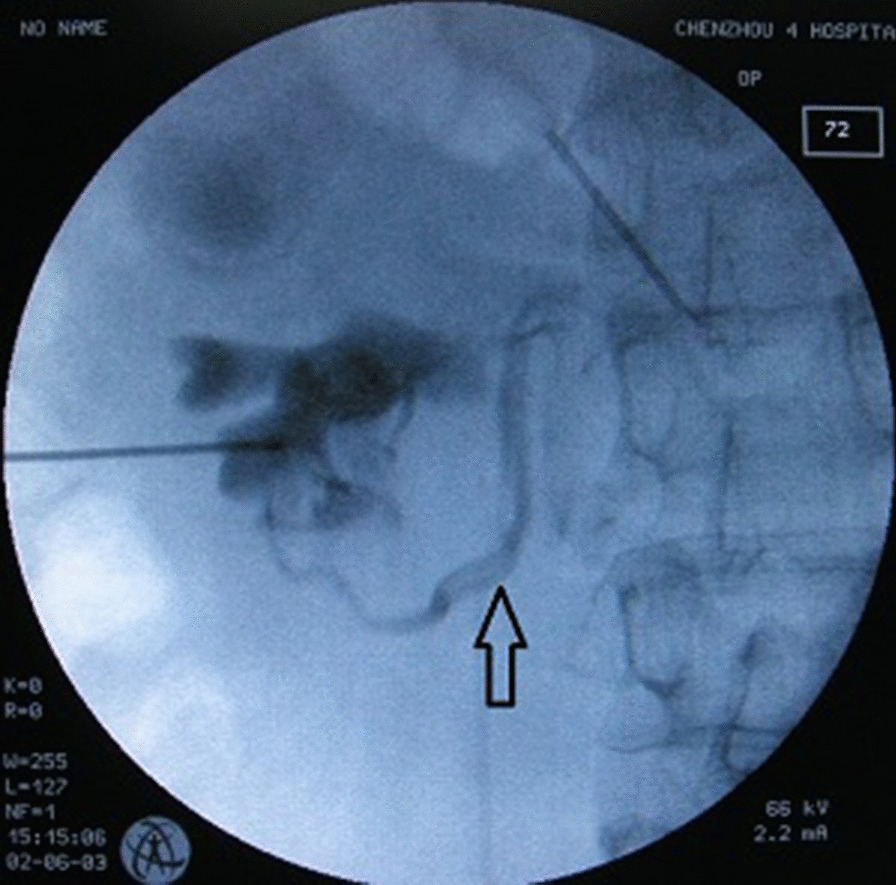
The radiograph of the kidneys, ureters and bladder revealing the left renal vein (arrow), the inferior calyx, and the suboptimal position of the puncture needle tip

Patient 2 was a 41-year-old woman who presented with left flank pain. Her medical history was unremarkable. White blood cells were detected in the urine. Empirical antibiotics were started. A CT scan of abdomen and pelvis showed the presence of left mild hydronephrosis and renal calculi was measured at 2.2 cm. The patient was diagnosed with renal calculi and mild hydronephrosis. A PCNL procedure protocol was planned. A ureteral catheter was inserted into the left ureter retrogradely. The target calix was the middle posterior calix. The puncture site of the target calyceal fornix was localized under fluoroscopy monitoring using contrast agent injected through ureteral catheter, producing a RP. Clear urine was seen on withdrawal of the stylet. During an injection of contrast agent in her left collecting system through the puncture needle, we were able to visualize her left renal vein filled with contrast agent (Fig. 3). We could also identify that the location of the puncture needle tip was not optimal. The puncture needle tip was near the calyceal infundibulum. The puncture needle was pulled out. A puncture tract passing through the middle posterior caliceal fornix was performed simultaneously and the renal calculi was removed uneventfully. The operative steps were as same as patient 1.

Among our two patients, we saw the renal vein filled with contrast agent during PCNL and identified the location of the puncture needle tip was not optimal. The puncture needle tip was located outside the collecting system near the calyceal infundibulum in the two patients. The visualization of renal vein branches during antegrade pyelography and bloody fluid drips confirmed the puncture needle tips located in a vein vessel or injured a vessel. The following procedures were not carried out. The original operation PCNL was performed successfully by another puncture tract in the two patients. They were discharged uneventfully with the stones cleaned by simultaneous PCNL.

Six articles relevant to the vein injury during PCN or PCNL were selected [3, 5–9]. The data of the six articles were summarized in Table 1. As noted in Table 1, the vein injury in patient undergoing PCN occurred in the initial stage of the operation. The guidewire or catheter perforated and migrated into the vein directly. In patient undergoing PCNL, the vein injury occurred either in the initial stage due to the dilator tearing motion or during the operation due to the fragmentation energy.

**Table 1:** Reports of mechanism of intravenous nephrostomy tube misplacement

First author	References	Original operation	Steps: first step	Steps: second step	Steps: third step	Is the guidewire in the vein?	When is the vein torn?
Dias-Filho	[3]	Tuber placement	A vein is perforated by a guidewire	The injured vein is dilated	The catheter is placed inside the vein	Yes	During dilating at the initial stage of operation
Kotb	[8]	Tuber placement	The silicon catheter passes into a major vein after penetrating the renal parenchymal			The guidewire is not used	At the initial stage of operation
Chen	[6]	PCNL	The fascial dilators tear a large vein	The sheath directs the catheter inside the vessels		No	During dilating at the initial stage of operation
Fu	[9]	PCNL	The dilator sheath injures the renal vein	The guidewire perforates into the renal vein and the injured vein is dilatated	The catheter is placed inside the vessels	Yes	During dilating at the initial stage of operation
Mazzucchi	[5]	PCNL	A lesion in a large renal vein branch is caused by the instruments	The sheath directs the catheter inside the vessels		No	During the fragmentation process
Wang	[7]	PCNL	A lesion in a large renal vein branch is caused by the instruments	The sheath directs the catheter inside the vessels		No	During the fragmentation process

## Discussion and conclusions

Intravenous tube misplacement is a rare complication of PCNL or PCN with the incidence of 0.02–0.05% [6, 9].

Three types of mechanism of intravenous tube misplacement were mentioned in literatures. The first type of mechanism was described by Chen et al. [6] and Fu et al. [9]. Firstly, the puncture needle passes through a branch of renal vein and its tip enter the collecting system. During inserting the guidewire through the puncture needle, the tip of puncture needle moves outside of the collecting system due to such factors as respiratory movement, narrow space between staghorn calculi and collecting system. The guidewire stays just outside the collecting system near the injured renal vein branch or perforates into the renal vein. If the misplaced improper location of guidewire is not recognized by the surgeon. Dilatation directed by the guidewire results in a torn lesion of the vein. Heavy venous bleeding occurs as soon as the dilator is removed. The nephrostomy tube migrates into the vascular system due to the prompt tube inserting and the direction of the sheath and\or the guidewire to the injured vein.

The second type of mechanism is as Mazzucchi et al. [5] and Wang et al. [7] describes. Firstly, the instruments used during surgery cause a lesion in a large branch of renal vein. Secondly, the sheath directs the nephrostomy tube inside the vascular system. The third mechanism can be observed in catheter placement [3, 8]. Firstly, the nephrostomy tube or the guidewire perforates a major branch of the renal vein. Then, the tube is placed inside the vessel directly, or guided by the guidewire.

Here we provide two patients with the imaging evidence and the scene of procedure demonstration to verify the renal vein injury in the first mechanism. In our patients 1 and 2 the location of the puncture needle tip was not optimal. We are able to visualize the renal vein and collecting system during an injection of contrast agent through the puncture needle. It indicates that the tip of puncture needle has pass through the branch of the renal vein and enters into the collecting system initially. However, during fluoroscopic imaging the tip of puncture needle moves outside of the collecting system. It stays near or inside the branch of the renal vein outside the collecting system. If the guidewire is inserted through the puncture needle, it will just stay outside the calix or perforate into the renal vein. Dilatation directed by the guidewire will result in the injury of the vein. The nephrostomy tube will migrate into the vascular system directed by the dilator sheath and\or the guidewire. To prevent such complication, review of previous imaging, including ultrasound, CT, or magnetic resonance imaging should be performed, to identify valuable anatomic information as to the optimal access entrance before establishing percutaneous access. The ideal entrance point should be along the avascular plane of Brodel in a position that is lateral to the renal calyx, and directed toward the renal pelvis. The puncture tract should pass through the caliceal fornix. After the tip of the needle is confirmed within the collecting system under ultrasound or fluoroscopy guidance. The tip of the needle should be maintained in the initial position with the needle depth no changing. Then, a guidewire is carefully and gently inserted into the renal collecting system. The guidewire should be also confirmed curling in the renal collecting system under ultrasound or fluoroscopy guidance.

In conclusion, visualization of the renal vein filled with contrast agent may be good evidence to verify the renal vein injury in patients with intravenous nephrostomy tube misplacement during PCNL or PCN. The suboptimal location of the puncture needle tip and visualization of the renal vein filled with contrast agent indicate the renal vein injury. One type of mechanism of intravenous nephrostomy tube misplacement is as following. Firstly, the guidewire stays outside the collecting system. Subsequently, dilatation directed by the guidewire results in the injury of the vein. Then, the nephrostomy tube migrates into the venous system due to prompt tube inserting and the direction of the sheath and\or the guidewire to the injured vein.

## Data Availability

Not applicable.

## Abbreviations

CT: Computed tomography
IVC: Inferior vena cava
PCNL: Percutaneous nephrolithotomy
PCN: Percutaneous nephrostomy
RP: Retrograde pyelography
